# Suppression of B-Raf(V600E) cancers by MAPK hyper-activation

**DOI:** 10.18632/oncotarget.7909

**Published:** 2016-03-04

**Authors:** Rawan Atiq, Rachel Hertz, Sophia Eldad, Elia Smeir, Jacob Bar-Tana

**Affiliations:** ^1^ Department of Human Nutrition and Metabolism, Hebrew University Medical School, Jerusalem, Israel 91120

**Keywords:** B-Raf(V600E), MAPK, melanoma, colorectal cancer, papillary thyroid carcinoma

## Abstract

B-Raf(V600E) activates MEK/MAPK signalling and acts as oncogenic driver of a variety of cancers, including melanoma, colorectal and papillary thyroid carcinoma. Specific B-Raf(V600E) kinase inhibitors (e.g., Vemurafenib) prove initial efficacy in melanoma followed shortly by acquired resistance, while failing in most other B-Raf(V600E) cancers due to primary resistance. Resistance is due to acquired mutations in the Ras/Raf/MEK/MAPK pathway and/or other oncogenic drivers that bypass B-Raf(V600E). Surprisingly, hyper-activation of MAPK by inhibiting its protein phosphatase 2A by a synthetic long-chain fatty acid analogue (MEDICA), results in oncogene-induced growth arrest and apoptosis of B-Raf(V600E) cancer cells. Growth arrest is accompanied by MAPK-mediated serine/threonine phosphorylation and suppression of a variety of oncogenic drivers that resist treatment by B-Raf(V600E) kinase inhibitors, including ErbB members, c-Met, IGFR, IRS, STAT3 and Akt. The combined activities of mutated B-Raf and MEDICA are required for generating hyper-activated MAPK, growth arrest and apoptosis, implying strict specificity for mutated B-Raf cancer cells.

## INTRODUCTION

B-Raf is a member of the Raf family of serine/threonine protein kinases. Mutated B-Raf kinases, like B-Raf(V600E), are potent activators of MEK/MAPK signalling, and drive oncogenic transformation in about 8% of all cancers, including 60%, 50%, 10% and 6% of papillary thyroid carcinoma (PTC), melanoma, colorectal cancer (CRC), and lung cancer, respectively [1]. B-Raf(V600E) tumors poorly respond to traditional chemotherapy, resulting in advancing targeted therapy with specific inhibitors that block B-Raf(V600E)/MEK/MAPK signalling (e.g., Vemurafenib/PLX4032, Dabrafenib) [1, 2]. These inhibitors show dramatic efficacy in melanoma, but essentially fail in most other B-Raf(V600E) cancers due to primary resistance [1]. Moreover, in spite of their initial efficacy in suppressing B-Raf(V600E) melanoma growth, acquired resistance develops shortly, resulting in melanoma relapse [1, 3]. Primary and/or acquired resistance to B-Raf(V600E) kinase inhibitors is due to mutations in the Ras/Raf/MEK/MAPK pathway that bypass B-Raf(V600E) [3–7]. These are complemented by a variety of de-repressed receptor tyrosine kinases (RTKs), including ErbB members, IGF1R and c-Met, that activate survival transduction pathways independently of B-Raf(V600E), including IRS/PI3K/Akt/mTORC1, C-Raf/MEK/MAPK, Jak/STAT3 and/or c-Src [7–15]. Current strategies to overcome resistance to B-Raf(V600E) kinase inhibitors employ a combination of B-Raf(V600E), MEK, Erk, ErbB, PI3K/Akt/mTORC1, JAK/STAT3 and/or c-Src inhibitors [1, 3]. However, resistance targets may dynamically evolve [1], and targeting each would require repeated biopsies upon relapse, as well as cost and safety considerations concerned with multiple drug treatment. Hence, counteracting mutated B-Raf(s) and the variety of individual oncogenic drivers that generate resistance to B-Raf(V600E)/MEK/MAPK inhibitors calls for an alternative treatment strategy.

Under certain cellular conditions, mutated B-Raf or Ras may drive oncogene-induced growth arrest rather than oncogenic transformation [16, 17]. Indeed, oncogene-induced growth arrest of pre-malignant B-Raf(V600E) naevi, blocks their progression to malignant metastatic melanoma [18]. Hence, forcing oncogene-induced growth arrest in mutated B-Raf cancers may offer a treatment mode that may avoid the chase after resistant oncogenic drivers that phase in/out in the course of suppressing mutated B-Raf by respective kinase inhibitors. AMP-activated protein kinase (AMPK) activators have indeed been reported to induce cellular growth arrest [19], implying their prospective use in suppressing B-Raf(V600E) cancers.

MEDICA analogs consist of long-chain, α,ω-dioic acids [HOOC-C(α′)-C(β′)-Q-C(β)-C(α)-COOH, where Q represents a long-chain core element], substituted in the αα′ (Mαα), ββ' (Mββ), and/or other optional core carbons [20, 21]. MEDICA analogs are not esterified into lipids, nor converted into ceramides, while their respective substitutions block their β-oxidation or esterification into lipids. MEDICA analogs induce mitochondrial ROS production [22] by gating the mitochondrial permeability transition pore [23], resulting in potent activation of AMPK [21]. ROS production and AMPK activation prompted our interest in studying MEDICA efficacy in the B-Raf(V600E) context.

## RESULTS

**Figure 1:**
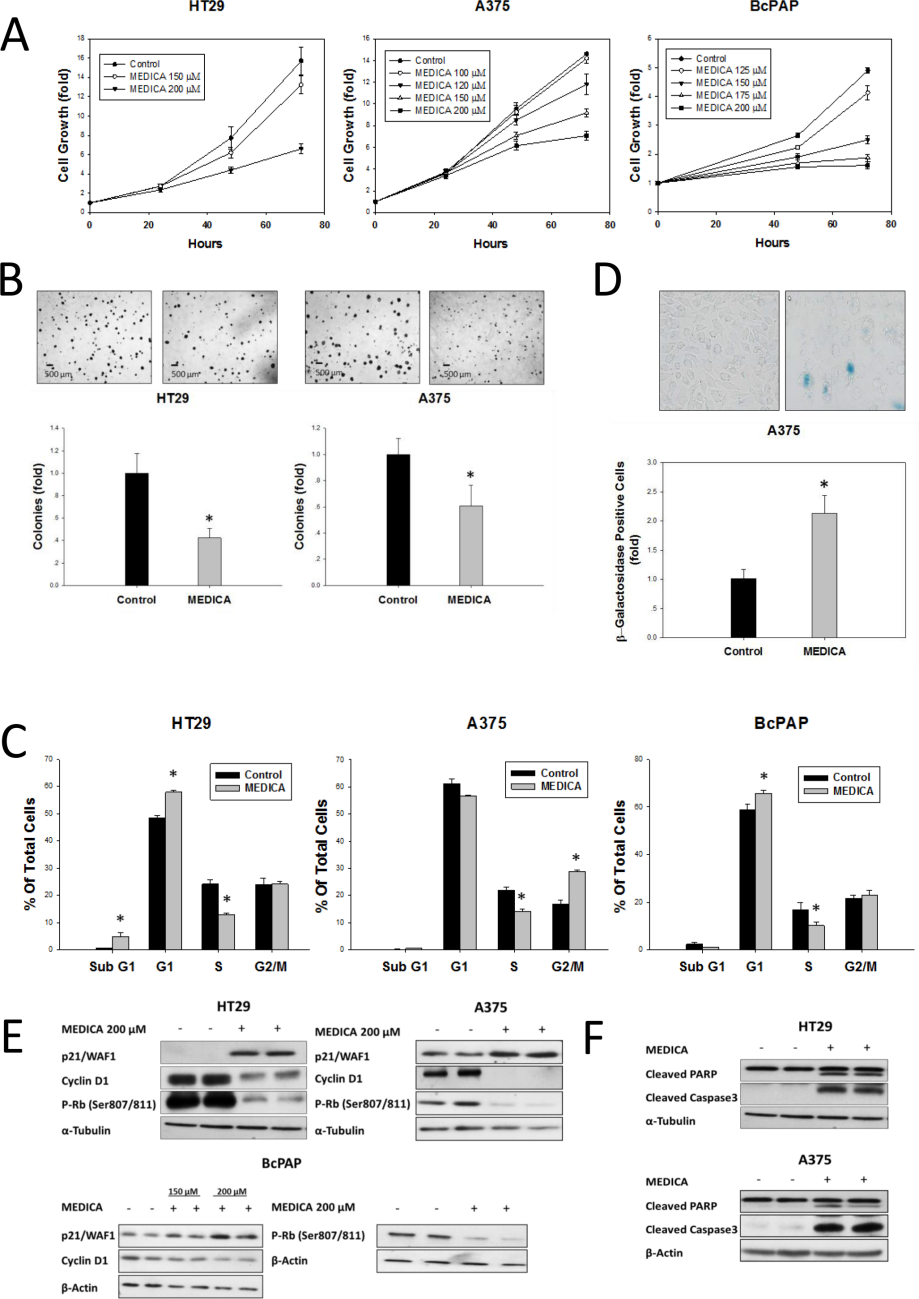
Growth Inhibition and apoptosis of B-Raf(V600E) cell types by MEDICA (**A–C**) Cell growth (A), anchorage-independent colonies (B), and cell cycle distribution (48 h) (C). Mean ± SD. *Significant as compared to control. Inset-representative micrographs. (**D**) Senescence-associated β-galactosidase. Mean ± SE. *Significant as compared to control. Inset-representative micrographs. (**E, F**) Cyclin D1, p21, P-Rb(Ser807/811), cleaved PARP and caspase 3. Representative blots.

Suppression of B-Raf(V600E)-driven transformation by MEDICA [HOOC-C(CH_3_)_2_ -(CH_2_)_12_-C(CH_3_)_2_-COOH] was verified in a variety of colorectal (CRC) (HT29, RKO), melanoma (A375, UACC62) and papillary thyroid carcinoma (PTC) (BcPAP) cells, where B-Raf(V600E) serves as major oncogenic driver, and treatment with B-Raf(V600E) kinase inhibitors fails due to primary or acquired resistance. MEDICA treatment resulted in suppressing cell proliferation and colony formation of the concerned cell types (Figure 1A, 1B). Growth suppression was accompanied by G0/G1 (HT29, BcPAP) or G2/M (A375) cell cycle arrest (Figure 1C), increase in senescence-associated β-galactosidase (Figure 1D), and pronounced increase in p21, with decrease in cyclin D1 and phospho-Rb(Ser807/811) (Figure 1E). Growth suppression was accompanied by cell apoptosis as verified by increase in cleaved PARP and caspase 3 (Figure 1F). The μM concentrations of MEDICA reflect its high binding affinity to serum albumin (higher than 99%, independently of MEDICA concentrations in the range of 0–0.9 mM), resulting in nM concentrations of the free MEDICA acid in the culture medium.

**Figure 2:**
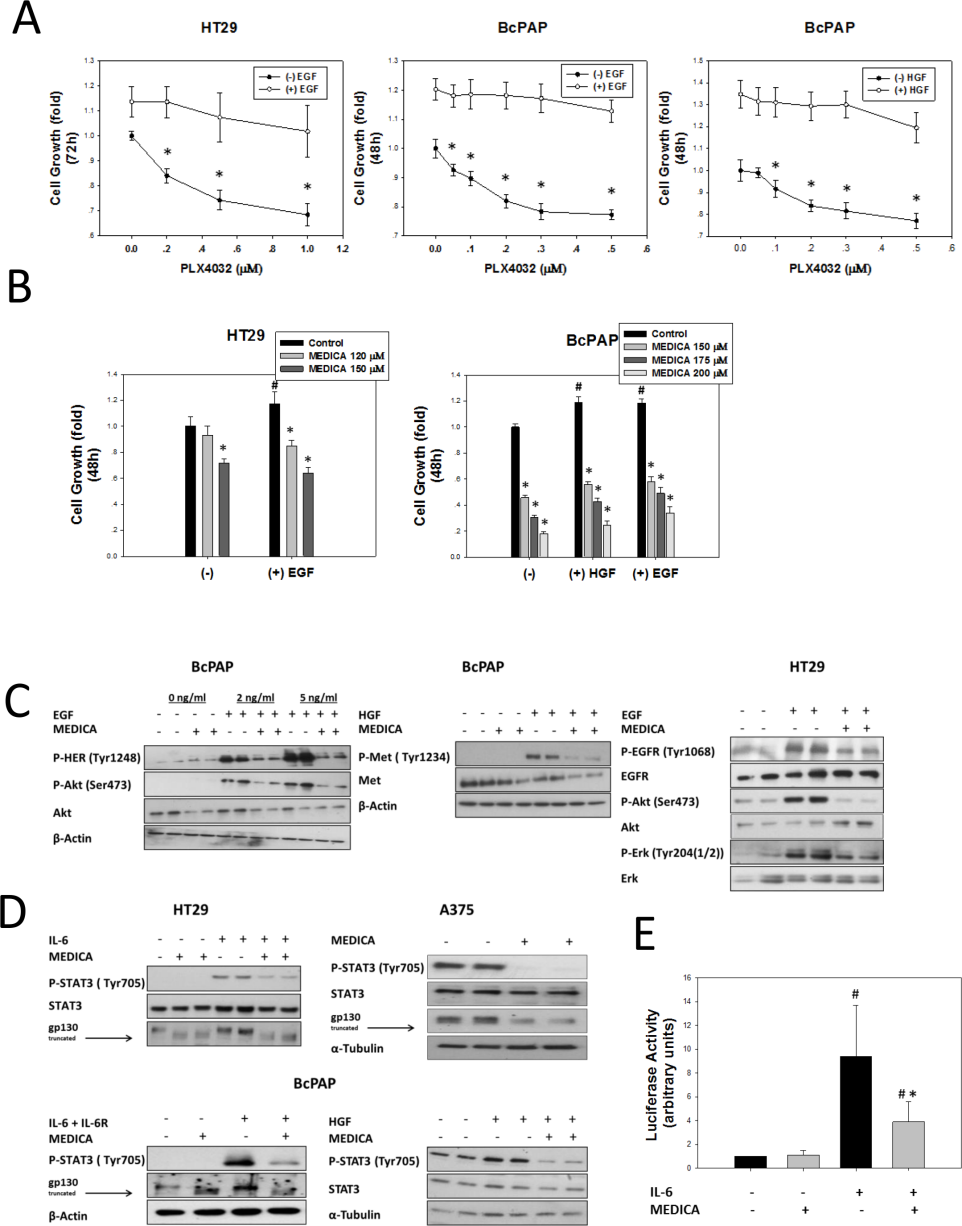
Suppression of PLX4032-resistant B-Raf(V600E) cell types by MEDICA (**A**) EGF- and HGF-induced resistance to PLX4032. Mean ± SD. *Significant as compared to control. (**B**) Suppression of PLX4032-resistant B-Raf(V600E) cell types by MEDICA. Mean ± SD. ^#^Significance of added cytokine as compared to its absence. *Significant as compared to respective control. (**C**) Suppression of EGF- and HGF-induced phosphorylation of EGFR, ErbB2/HER, MET, Erk and Akt by MEDICA. Representative blots. (**D**) Suppression of gp130, IL6- and HGF-induced phosphorylation of STAT3, and constitutive STAT3 by MEDICA. Representative blots. (**E**) Suppression of transfected STAT3 reporter by MEDICA (HT29 cells). Mean ± SE. ^#^Significant as compared to control. *Significant as compared to control IL-6.

Growth and survival of B-Raf(V600E) cancers is driven by B-Raf(V600E)-activated MAPK, as well as by a variety of de-repressed RTKs and transduction pathways that resist B-Raf(V600E) kinase inhibitors, including ErbB members, IGF1R, c-Met, IRS/PI3K/Akt/mTORC1, C-Raf/MEK/MAPK, Jak/STAT3 and/or c-Src [7–15]. In agreement with previous reports, resistance to B-Raf(V600E) inhibitors is exemplified here in CRC HT29 and PTC BcPAP cells by lack of response to PLX4032 under conditions of EGF- or HGF-activated cell growth (Figure 2A). In contrast, EGF- or HGF-activated growth of HT29 and BcPAP cells was suppressed by MEDICA (Figure 2B), with decrease in EGF-activated phospho-EGFR(Tyr1068), phospho-ErbB2(Tyr1248), phospho-Akt(Ser473) and phospho-Erk(Tyr204), as well as in HGF-activated phospho-Met(Tyr1234) (Figure 2C). In line with that, MEDICA suppressed the IL-6- and HGF-activated phospho-STAT3(Tyr705) in CRC HT29 or PTC BcPAP cells, and the constitutively-active STAT3 in A375 melanoma cells (Figure 2D). STAT3 suppression by MEDICA was further verified in CRC HT29 cells transfected with STAT3 reporter gene (Figure 2E). STAT3 suppression by MEDICA was accompanied by increase in truncated gp130 (Figure 2D).

**Figure 3:**
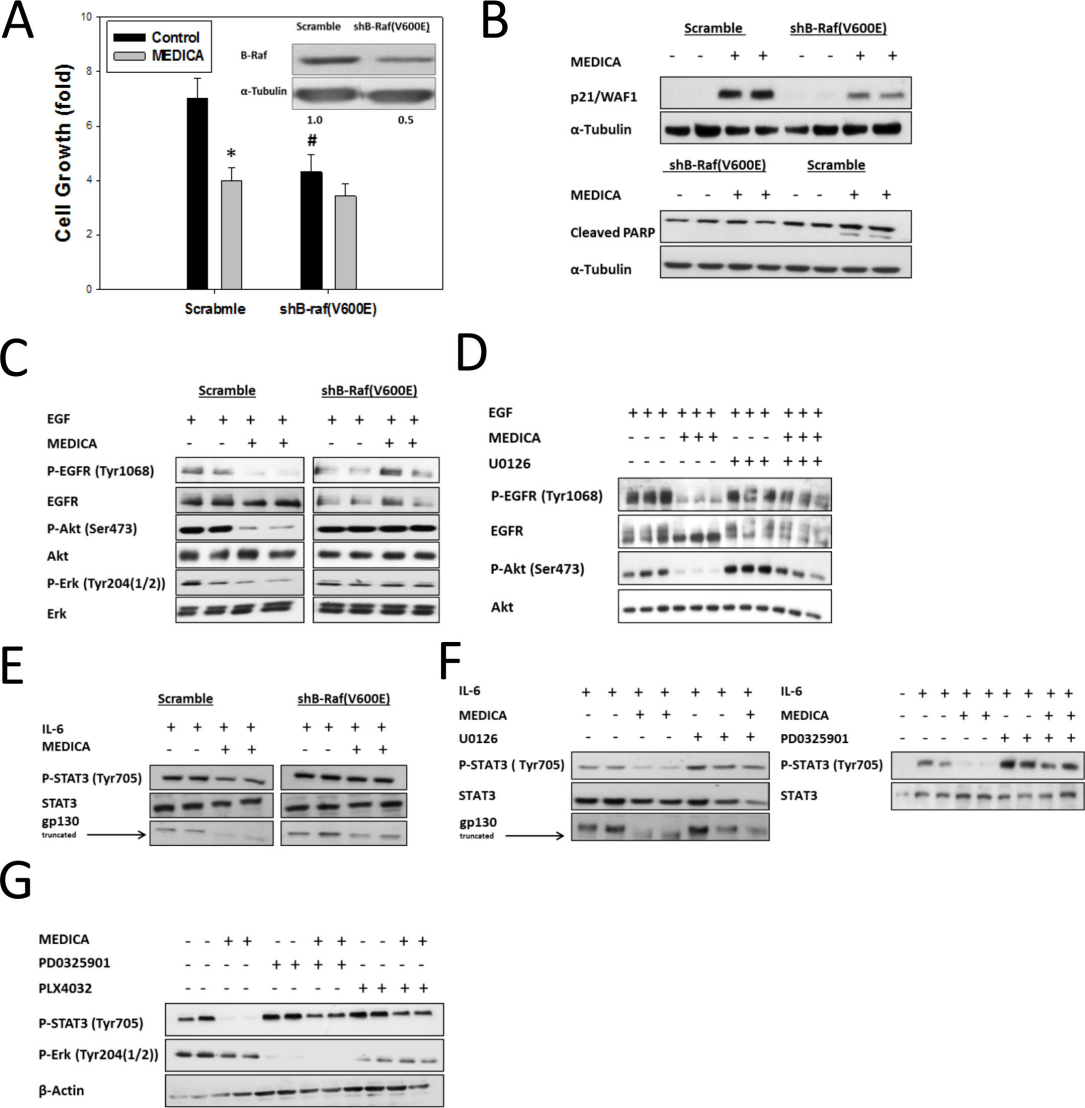
Growth suppression of B-Raf(V600E) cells by MEDICA is B-Raf(V600E)-dependent (**A**) Suppression of HT29 cell growth (48 h) by MEDICA is abrogated by infected shB-Raf(V600E). Mean ± SD. *Significant as compared to respective control. ^#^Significant as compared to respective scramble. (**B**) MEDICA-induced p21 and cleaved PARP in HT29 cells is abrogated by infected shB-Raf(V600E). (**C**) Suppression of EGF-activated EGFR, Akt and Erk in HT29 cells by MEDICA is abrogated by infected shB-Raf(V600E). (**D**) Suppression of EGF-activated EGFR and Akt in HT29 cells by MEDICA is abrogated by inhibiting MEK by U0126. (**E, F**) Suppression of IL-6-activated STAT3 in HT29 cells by MEDICA is abrogated by infected shB-Raf(V600E), or upon inhibiting MEK by U0126 or PD0325901. (**G**) Suppression of constitutive STAT3 and Erk in A375 cells by MEDICA is abrogated by inhibiting B-Raf(V600E) by PLX4032, or by inhibiting MEK by PD0325901. (B-G) Representative blots.

Surprisingly, suppression of B-Raf(V600E) cancers by MEDICA strictly depended on maintaining the B-Raf(V600E)/MEK/MAPK activity. Thus, MEDICA failed to suppress growth (Figure 3A), or to increase cellular p21 and cleaved PARP (Figure 3B), in CRC HT29 cells infected with shB-Raf(V600E). Also, suppression of EGF-activated phospho-EGFR(Tyr1068), phospho-Akt(Ser473) and phospho-Erk(Tyr204) by MEDICA was abrogated in CRC HT29 cells infected with shB-Raf(V600E) (Figure 3C), or by inhibiting MEK activity by added U0126 (Figure 3D). Similarly, suppression of IL-6-induced phospho-STAT3(Tyr705) and truncation of gp130 by MEDICA were abrogated in CRC HT29 cells infected with shB-Raf(V600E) (Figure 3E), or by inhibiting MEK activity by either U0126 or PD325901 (Figure 3F). Similarly, suppression of constitutive STAT3 in A375 melanoma cells by MEDICA was abrogated by inhibiting B-Raf(V600E) kinase by PLX4032, or by inhibiting MEK activity by PD325901 (Figure 3G). The obligatory requirement for maintaining the B-Raf(V600E)/MEK/MAPK activity in enabling growth arrest of B-Raf(V600E) cancers by MEDICA, may indicate a requirement for hyper-activation of MAPK/Erk by the combined activities of B-Raf(V600E) and MEDICA. In line with that, our further studies focused on the mode of basal Erk activation by MEDICA.

**Figure 4:**
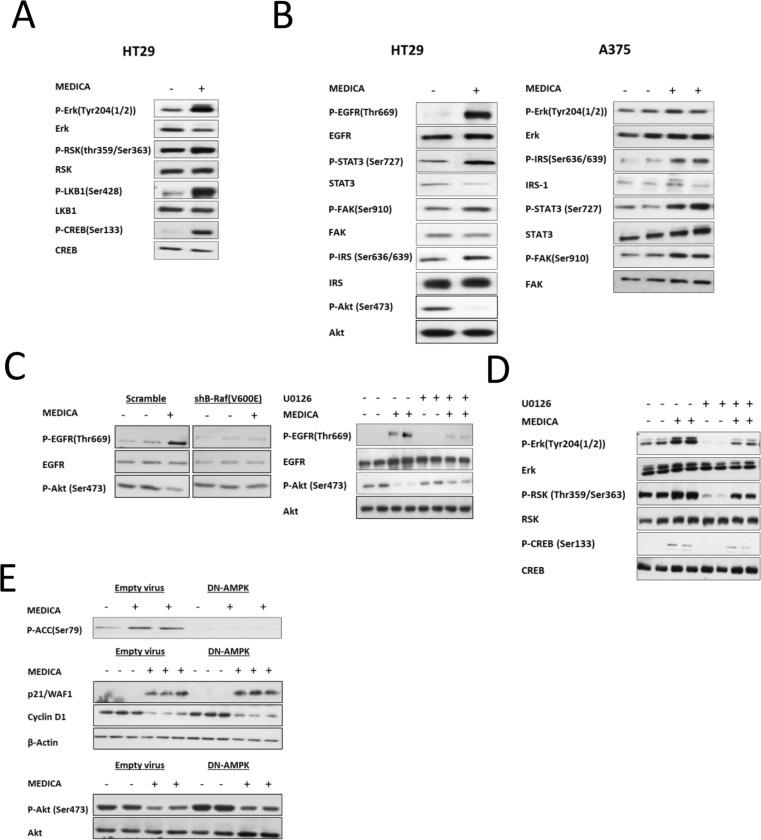
Erk activation by MEDICA (**A, B**) MEDICA activates Erk and its downstream targets RSK(Thr359/Ser363), LKB1(Ser428), CREB(Ser133), EGFR(Thr669), STAT3(Ser727), FAK(Ser910) and IRS1(Ser636/639), while inhibiting basal Akt(Ser473). (**C**) shRNA B-Raf(V600E) or U0126 abrogates EGFR(Thr669) phosphorylation and inhibition of basal Akt(Ser473) by MEDICA (HT29 cells). (**D**) Activation of Erk and its canonical downstream targets by MEDICA is MEK-independent (HT29 cells). (**E**) Growth suppression, Akt inhibition and apoptosis by MEDICA are AMPK-independent (HT29 cells). (A-E) Representative blots.

Erk activation by MEDICA was verified in terms of increase in phospho-Erk(Tyr204) as well as phosphorylation of Erk downstream canonical targets, including p90RSK, LKB1 and CREB (Figure 4A). Most importantly, in addition to phosphorylation of Erk canonical targets, MEDICA-activated Erk resulted in serine/threonine phosphorylation of Erk downstream receptor tyrosine kinases (RTKs) and transduction elements that serve as oncogenic drivers of resistant B-Raf(V600E) cancers, including EGFR(Thr669), STAT3(Ser727), FAK(Ser910) and IRS1(Ser636/639), while suppressing basal phospho-Akt(Ser473) (Figure 4B). The phosphorylation of Erk targets by MEDICA required the combined activities of B-Raf(V600E) and MEDICA, being abrogated in cells transfected with shB-Raf(V600E), or by inhibiting MEK activity by U0126 (Figure 4C). The requirement for hyper-activated Erk in enabling both, the serine/threonine phosphorylation of EGFR(Thr669), STAT3(Ser727), IRS1(Ser636/639) and FAK(Ser910), and the abrogation of their respective phospho-EGFR(Tyr1068), phospho-STAT3(Tyr705), phospho-Akt(Ser473) and p21 targets (Figures 2, 3), may indicate that serine/threonine phosphorylation by hyper-activated Erk is causal for silencing the concerned survival drivers. Of note, activation of Erk and its downstream targets by MEDICA was maintained under conditions of inhibiting MEK by U0126 (Figure 4D), implying that activation of Erk by MEDICA was MEK-independent, but may reflect stabilization of phospho-Erk(Tyr204). Also, increase in p21, decrease in cyclin D1, and suppression of EGF-activated phospho-Akt(Ser473) by MEDICA, were all maintained upon inhibiting AMPK activity by dominant-negative AMPK (Figure 4E), implying that MEDICA effects were AMPK-independent.

**Figure 5:**
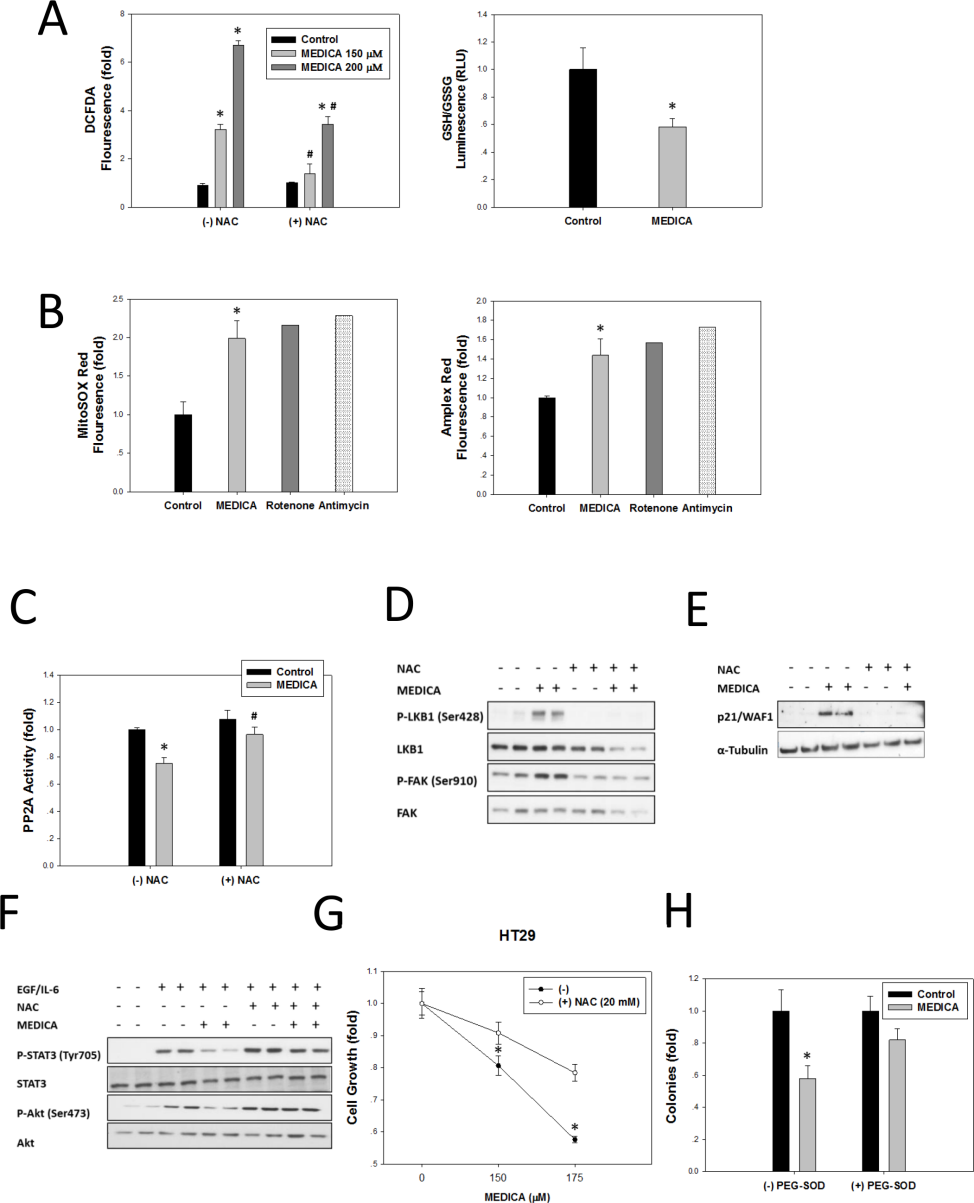
MEDICA-induced ROS production in HT29 cells (**A**) MEDICA-induced ROS production as verified by DCFDA fluorescence and decrease in GSH/GSSG ratio. Mean ± SE. *Significant as compared to respective control. ^#^Significant as compared to the respective MEDICA concentrations. (**B**) MEDICA-induced mitochondrial ROS production as verified by mitoSOX and succinate-dependent Amplex Red fluorescence. Mean ± SD. *Significant as compared to control. (**C**) Suppression of PP2A activity by MEDICA-induced ROS. Mean ± SE. *Significant as compared to respective control. ^#^Significant as compared to respective control. (**D–F**) Activation of Erk downstream targets (D), increase in p21 (E), and suppression of EGF- and IL-6-induced STAT3 and Akt (F) by MEDICA are abrogated by NAC. Representative blots. (**G**) Suppression of cell growth (48 h) by MEDICA is abrogated by NAC. Mean ± SD. *Significant as compared to growth in NAC absence. (**H**) Suppression of clonogenic growth by MEDICA is abrogated by PEG-SOD (100 u/ml). Mean ± SD. *Significant as compared to respective control.

Phospho-Erk(Tyr204) stability depends on its dephosphorylation by protein phosphatases, including PP2A [24]. Erk phosphatases are inhibited by reactive oxygen species (ROS) that may oxidize their respective catalytic cysteines [25, 26]. MEDICA-induced ROS production was verified in CRC HT29 cells by increase in DCFDA fluorescence, being abrogated by added N-acetyl cysteine (NAC), with decrease in GSH/GSSG, (Figure 5A). Mitochondrial ROS production by MEDICA was verified by increase in mitoSOX and in succinate-induced Amplex Red fluorescence, being similar to that of rotenone or antimycin (Figure 5B). MEDICA effects were still maintained in the presence of diphenyleneiodonium (not shown), indicating that MEDICA-induced ROS production was not mediated by NADPH oxidase activation. MEDICA-induced ROS resulted in suppressing PP2A activity, being abrogated by added NAC (Figure 5C). Most importantly, added NAC resulted in nullifying Erk phosphorylation of LKB1(Ser428) and FAK(Ser910) (Figure 5D), in abrogating MEDICA-induced p21 (Figure 5E), and in resuming EGF-induced phospho-Akt(Ser473) and IL6-induced phospho-STAT3(Tyr705) (Figure 5F). Inhibition of ROS production by NAC or PEG-SOD further resulted in abrogating suppression of CRC HT29 cell growth (Figure 5G) and HT29 colony formation (Figure 5H) by MEDICA. Hence, Erk activation by B-Raf(V600E)/MEK, combined with suppression of its de-phosphorylation by MEDICA-induced ROS, may account for Erk hyper-activation, resulting in growth arrest and apoptosis of B-Raf(V600E) cancer cells.

## DISCUSSION

Treatment of a variety of B-Raf(V600E) cancer cells by MEDICA, including PTC, CRC and melanoma, is shown here to result in oncogene-induced growth inhibition, followed by apoptosis. Growth arrest is accompanied by suppressing survival RTKs, including ErbB members and c-Met, as well as their STAT3, IRS and Akt transducers that serve as resistance nodes upon treating B-Raf(V600E) cancers with B-Raf(V600E) kinase inhibitors. Suppression of survival RTKs and their transducers may be ascribed to their serine/threonine phosphorylation by hyper-activated Erk, resulting in their degradation or in suppressing their oncogenic activity [27–33]. Similarly, FAK(Ser910) phosphorylation by hyper-activated Erk may account for MEDICA-induced increase in p21 [34, 35].

MEDICA effects are all accounted for by hyper-activated Erk, generated due to its basal increased activity due to mutated B-Raf, being further intensified by suppressing Erk PP2A phosphatase by MEDICA-induced ROS. Erk hyper-activation by MEDICA in the mutated B-Raf context is further driven by lack of feedback inhibition of the mutated B-Raf by activated Erk [3]. Of note, the combined activities of mutated B-Raf and MEDICA-induced ROS are obligatory in generating hyper-activated Erk and its growth arrest outcome, since suppressing B-Raf(V600E)/MEK kinase activities by shB-Raf(V600E) and/or by MEK inhibitors, or suppressing MEDICA-induced ROS by antioxidants, results in resuming survival RTKs, their respective transduction pathways, and cell growth. The obligatory requirement for the combined activities of mutated B-Raf and MEDICA-induced ROS in inducing hyper-activated Erk, implies strict specificity towards mutated B-Raf cancer cells.

Growth inhibition by hyper-activated Erk due to inhibited PP2A may imply that canonical oncogenes and tumor suppressors may exchange roles in the mutated B-Raf cellular context, with the Erk oncogene playing the role of a tumor suppressor, and the PP2A tumor suppressor acting as oncogene. Growth inhibition by hyper-activated Erk may also account for the recently proposed “drug holiday” treatment protocol for resistant B-Raf(V600E) tumors [36, 37], whereby intermittent elimination of the B-Raf(V600E) kinase inhibitor may result in Erk hyper-activation leading to transient suppression of resistant targets.

Growth arrest and suppression of survival oncogenic drivers by hyper-activated Erk may offer a treatment mode for mutated B-Raf cancers, that avoids the frustrating chase after individual resistant targets that dynamically evolve upon suppressing B-Raf(V600E)/MEK/MAPK by kinase inhibitors. If you can't beat mutated B-Raf, join it. Admittedly, growth inhibition by hyper-activated Erk is threatened by cellular events that may down regulate B-Raf(V600E)/MEK/MAPK activity [38], activate the PI3K/Akt/mTORC1 transduction pathway [39], or interfere with senescence maintenance [40], resulting in resuming oncogenesis. However, the natural sequel of most naevi may indicate that once established, oncogene-induced growth arrest may be maintained throughout life.

## MATERIALS AND METHODS

### Cultured cells

Human colorectal cancer (HT29, RKO; ATCC) and melanoma (A375, UACC62) cell lines were cultured in DMEM (Biological Industries, Beit Haemek, Israel) supplemented with 10% fetal calf serum, 2 mM L-glutamine, and penicillin/streptomycin solution at 37°C in humidified atmosphere containing 5% CO2. Papillary thyroid carcinoma cells (BCPAP) were cultured in RPMI1640 supplemented with 10% fetal calf serum, 10 mM HEPES (pH 7.4), 1 mM pyruvate, and penicillin/streptomycin solution. Cells were cultured for 16–48 h with MEDICA (200–250 μM, unless otherwise indicated), N-acetylcysteine (5–20 mM unless otherwise indicated) or growth factors (EGF (6 ng/ml), HGF (6 ng/ml)) as indicated. Transduction pathways were induced by adding EGF (50–100 ng/ml), HGF (5 ng/ml), IL-6 (20 ng/ml) or IL-6R (500 ng/ml) as indicated. EGF and HGF were added during the last 10–15 min of incubation. IL-6 and IL-6R were added during the last 30 min of incubation. MEK inhibitors (U0126 (20 μM) or PD0325901 (50 nM)) were added 30 min prior to MEDICA.

### Cell growth

Assayed by methylene blue.

### Soft agar assay

0.5% bottom and 0.3% top agar plates were plated with culture medium containing 0.3% agar and 5000 cells with additions as indicated. Following 13 d, anchorage-independent colonies exceeding 100 microns in size were counted.

### Clonogenic assay

Following treatment for 24 h, cells were trypsinized, plated on 60 mm plates, were allowed to form colonies for 10 d, then fixed and stained with methylene blue. Colonies exceeding 500 microns in size were counted.

### Transient transfection

HT29 cells cultured to 70% confluence were transfected with reporter plasmid for STAT3 [M67-TATA-TK-LUC] (0.3 μg) together with β-galactosidase plasmid [CMV-β-galactosidase] (0.05 μg) using JetPEI (PolyPlus Transfection, #101–10). Following 9 h, culture medium was replaced with serum free DMEM containing 0.2% fatty acid free BSA supplemented with MEDICA for 19 h as indicated. IL-6 (20 ng/ml) was added during the last 6 h. Luciferase activity (Promega #E4030) was normalized to β-galactosidase activity (Bio Rad, #170–3150).

### Lentivirus infection

(V600E)-specific shB-Raf plasmid (nucleotides 1853–1871 (NM_004333)) and control shRNA were from H. Rizos (Westmead Institute for Cancer Research, Australia) and R. Marais (Cancer Research, Manchester Institute, UK). HT29 cells infected with variable viral titers were monitored for GFP expression, and cells with 90% infection yield were further used. HT29 cells were infected with variable DN-AMPKα1(D157A) [21] viral titers and infection efficiency was monitored by phospho-ACC(Ser79).

### Senescence-associated β-galactosidase

Cells cultured on coverslip were fixed with (1% Formaldehyde, 0.2% Glutaraldehyde in PBS pH 7.4), washed once with PBS and twice with PBS/1 mM MgCl2 (pH 6.0). Cells were stained with x-gal solution (1 mM MgCl2, 5 mM K3Fe(CN)6, 5 mM K4Fe(CN)6 3H2O and 1 mg/ml x-gal) at 37°C overnight and washed three times with PBS. Cells were counterstained with DAPI and mounted. Senescent cells were counted vs. DAPI using the Live-imaging Program.

### Cell lysis and Western blotting

Cultured cells were scraped with lysis buffer (50 mM Tris HCl pH 8.0, 1% Triton X-100, 1 mM EGTA, 1 mM EDTA, 150 mM NaCl, 5 mM NaPPi, 50 mM NaF, 1 mM PMSF, 1 mM Na Vanadate, 40 nM bpVfan and protease inhibitor cocktail (Sigma)), and centrifuged for 15 min at 12,500 rpm. Protein concentration was determined by BCA (Thermo Scientific). Protein lysates were prepared in SDS sample buffer (62 mM Tris (pH 6.8), 2.3% SDS, 0.64 mM mercaptoethanol, 10% glycerol), subjected to SDS-PAGE, electro transferred onto cellulose nitrate membranes (Schleicher & Schuell, Dassel, Germany) and immunoblotted with the indicated first antibody, followed by HRP-labeled second antibody.

### Cell cycle distribution

Cells were trypsinized, washed with cold PBS, suspended in PBS/70% ethanol, and kept at −20°C. For FACS analysis, cells were centrifuged, washed with PBS and suspended in 700 μl propidium iodide (PI)/Triton X-100/RNAase A staining solution (20 μg/ml PI, 0.1% Triton X-100, 0.1 mg/ml RNAase A in PBS). Cell cycle distribution was analyzed using FACScan (BD Biosciences).

### ROS production

ROS production was determined by 2,7 dichlorofluoresceine diacetate (DCFDA) (5 μM) added to respective cell cultures for the last 15 min of incubation. Cells were washed once with PBS, and lysed with 0.5% TX-100. DCFDA fluorescence was determined by 485/530 nm excitation/emission analysis. Glutathione (GSH)/Glutathione disulfide (GSSG) ratio was determined by the GSH/GSSG-Glo assay kit (Promega).

### Succinate-dependent mitochondrial ROS production

Cells (0.3 mg/ml protein) were permeabilized with 40 μg/ml digitonin, followed by adding 20 μM MEDICA, 1 μM rotenone or 10 μM antimycin as indicated. Succinate-dependent ROS was measured in the presence of 10 mM HEPES buffer consisting of 10 mM succinate, 120 mM KCL, 5 mM KH_2_PO_4,_ 5 mM EGTA, 5 mM MgCl_2_, 1 μM Amplex red and 20 U/ml HRP. Amplex red oxidation was monitored by 560/590 excitation/emission fluorescence.

### Mitochondrial superoxide production

Cells treated with 200 μM MEDICA for 5 h were trypsinized, resuspended in phenol free medium and further incubated for 30–120 min with MEDICA, followed by adding 3.3 μg/ml MitoSOX (in DMSO) for the last 30 min. Cell were analyzed using FACScan (BD Biosciences). Rotenone (2 μM) and Antimycin (0.75 μM) were added as positive controls where indicated.

### PP2A activity assay

Cells were washed once with cold PBS, lysed with lysis buffer (20 mM Imidazole pH 7.2, 2 mM EDTA, 1 mM EGTA, 1 mM PMSF, and protease inhibitor cocktail (Sigma)), sonicated for 10 sec and centrifuged at 2000 g at 4°C for 5 min. 250 μg protein lysate was incubated with 2.5 μg PP2A antibody and Sepharose A/G beads (Pierce Biotechnology) for 2 hours at 4°C with rotation. Beads were washed with TBS, then by assay buffer (100 μM CaCl2, 50 mM Tris-HCL pH 7.0), and re-suspended in 50 μl reaction mix containing 10 μl assay buffer and 200 μM phosphate substrate peptide (RRA(pT)VA). Samples were incubated at 30°C for 30 min. Reaction was stopped with 50 μl Molybdate Dye Solution (Promega #V2460), incubated for 15 min at room temperature and absorbance determined at 630 nm.

### Materials

MEDICA [α,α′-tetramethyl hexadecanedioic acid, HOOC-C(CH_3_)_2_ -(CH_2_)_12_-C(CH_3_)_2_-COOH] was synthesized as previously described [21]. Anti-p21/WAF1 (#2946), anti-Cyclin D1(#2922), anti-phospho-RB(#9308), anti-β-Actin (#4967), anti-PARP (#9542), anti-cleaved Caspase3 (#9661), anti-phospho-Akt(Ser473) (#4060), anti-Akt (#9272), anti-phospho-STAT3(Tyr705) (#9145), anti-phospho-EGFR(Thr669) (#3056), anti-phospho-LKB1(Ser428) (#3051), anti-LKB1(#3050), anti-phospho-CREB(Ser133) (#9191), anti-CREB (#9197), anti-phospho-IRS-1(Ser636/639) (#2388), anti-phospho-ACC(Ser79)(#3661) antibodies were from Cell Signaling. Anti-EGFR(sc-03), anti-phospho-Erk(Tyr204) (sc-7383), anti-Erk (sc-93), anti-Met (sc-10), anti-STAT3 (sc-8019), anti-phospho-RSK1/2(Thr359/Ser363) (sc-12898), anti-RSK(sc-231), anti-phospho-STAT3(Ser727) (sc-8001), anti-IRS-1(sc-559) antibodies were from Santa Cruz Biotechnology. Anti-phospho-EGFR(Tyr1068) (ab40815), anti-phospho-HER(Tyr1248) (ab47755) antibodies were from Abcam. Anti-phospho-FAK(Ser910) (#445596) antibody was from Invitrogen. Anti-FAK(#61087) antibody was from BD. Anti-gp130 (#29731) antibody was from Upstate Biotechnology. Anti-α-Tubulin (T6074) antibody was from Sigma. EGF and HGF were from PeproTech. IL6 and IL6R were from R & D Systems. U0126 and PD0325901 were from Sigma.

### Statistics

Statistics was performed by two-tailed repeated measure analysis of variance (GraphPad). Significance (*P* < 0.05) was analyzed by unpaired *t*-test with Welch correction.

## References

[1] Holderfield M, Deuker MM, McCormick F, McMahon M (2014). Targeting RAF kinases for cancer therapy: BRAF-mutated melanoma and beyond. Nat Rev Cancer.

[2] Joseph EW, Pratilas CA, Poulikakos PI, Tadi M, Wang W, Taylor BS, Halilovic E, Persaud Y, Xing F, Viale A, Tsai J, Chapman PB, Bollag G (2010). The RAF inhibitor PLX4032 inhibits ERK signaling and tumor cell proliferation in a V600E BRAF-selective manner. Proc Natl Acad Sci U S A.

[3] Lito P, Rosen N, Solit DB (2013). Tumor adaptation and resistance to RAF inhibitors. Nat Med.

[4] Poulikakos PI, Persaud Y, Janakiraman M, Kong X, Ng C, Moriceau G, Shi H, Atefi M, Titz B, Gabay MT, Salton M, Dahlman KB, Tadi M (2011). RAF inhibitor resistance is mediated by dimerization of aberrantly spliced BRAF(V600E). Nature.

[5] Johannessen CM, Boehm JS, Kim SY, Thomas SR, Wardwell L, Johnson LA, Emery CM, Stransky N, Cogdill AP, Barretina J, Caponigro G, Hieronymus H, Murray RR (2010). COT drives resistance to RAF inhibition through MAP kinase pathway reactivation. Nature.

[6] Pratilas CA, Taylor BS, Ye Q, Viale A, Sander C, Solit DB, Rosen N (2009). (V600E)BRAF is associated with disabled feedback inhibition of RAF-MEK signaling and elevated transcriptional output of the pathway. Proc Natl Acad Sci U S A.

[7] Nazarian R, Shi H, Wang Q, Kong X, Koya RC, Lee H, Chen Z, Lee MK, Attar N, Sazegar H, Chodon T, Nelson SF, McArthur G (2010). Melanomas acquire resistance to B-RAF(V600E) inhibition by RTK or N-RAS upregulation. Nature.

[8] Montero-Conde C, Ruiz-Llorente S, Dominguez JM, Knauf JA, Viale A, Sherman EJ, Ryder M, Ghossein RA, Rosen N, Fagin JA (2013). Relief of feedback inhibition of HER3 transcription by RAF and MEK inhibitors attenuates their antitumor effects in BRAF-mutant thyroid carcinomas. Cancer Discov.

[9] Corcoran RB, Ebi H, Turke AB, Coffee EM, Nishino M, Cogdill AP, Brown RD, Della Pelle P, Dias-Santagata D, Hung KE, Flaherty KT, Piris A, Wargo JA (2012). EGFR-mediated re-activation of MAPK signaling contributes to insensitivity of BRAF mutant colorectal cancers to RAF inhibition with vemurafenib. Cancer Discov.

[10] Liu F, Cao J, Wu J, Sullivan K, Shen J, Ryu B, Xu Z, Wei W, Cui R (2013). Stat3-targeted therapies overcome the acquired resistance to vemurafenib in melanomas. J Invest Dermatol.

[11] Girotti MR, Pedersen M, Sanchez-Laorden B, Viros A, Turajlic S, Niculescu-Duvaz D, Zambon A, Sinclair J, Hayes A, Gore M, Lorigan P, Springer C, Larkin J (2013). Inhibiting EGF receptor or SRC family kinase signaling overcomes BRAF inhibitor resistance in melanoma. Cancer Discov.

[12] Turke AB, Song Y, Costa C, Cook R, Arteaga CL, Asara JM, Engelman JA (2012). MEK inhibition leads to PI3K/AKT activation by relieving a negative feedback on ERBB receptors. Cancer Res.

[13] Villanueva J, Vultur A, Lee JT, Somasundaram R, Fukunaga-Kalabis M, Cipolla AK, Wubbenhorst B, Xu X, Gimotty PA, Kee D, Santiago-Walker AE, Letrero R, D'Andrea K (2010). Acquired resistance to BRAF inhibitors mediated by a RAF kinase switch in melanoma can be overcome by cotargeting MEK and IGF-1R/PI3K. Cancer Cell.

[14] Xing M (2007). BRAF mutation in papillary thyroid cancer: pathogenic role, molecular bases, and clinical implications. Endocr Rev.

[15] Logue JS, Morrison DK (2012). Complexity in the signaling network: insights from the use of targeted inhibitors in cancer therapy. Genes Dev.

[16] Cagnol S, Chambard JC (2010). ERK and cell death: mechanisms of ERK-induced cell death—apoptosis, autophagy and senescence. FEBS J.

[17] Park JI (2014). Growth arrest signaling of the Raf/MEK/ERK pathway in cancer. Front Biol (Beijing).

[18] Michaloglou C, Vredeveld LC, Soengas MS, Denoyelle C, Kuilman T, van der Horst CM, Majoor DM, Shay JW, Mooi WJ, Peeper DS (2005). BRAFE600-associated senescence-like cell cycle arrest of human naevi. Nature.

[19] Jones RG, Plas DR, Kubek S, Buzzai M, Mu J, Xu Y, Birnbaum MJ, Thompson CB (2005). AMP-activated protein kinase induces a p53-dependent metabolic checkpoint. Mol Cell.

[20] Bar-Tana J, Ben-Shoshan S, Blum J, Migron Y, Hertz R, Pill J, Rose-Khan G, Witte EC (1989). Synthesis and hypolipidemic and antidiabetogenic activities of beta,beta,beta',beta'-tetrasubstituted, long-chain dioic acids. J Med Chem.

[21] Za'tara G, Bar-Tana J, Kalderon B, Suter M, Morad E, Samovski D, Neumann D, Hertz R (2008). AMPK activation by long chain fatty acyl analogs. Biochem Pharmacol.

[22] Gluschnaider U, Hertz R, Ohayon S, Smeir E, Smets M, Pikarsky E, Bar-Tana J (2014). Long-chain fatty acid analogues suppress breast tumorigenesis and progression. Cancer Res.

[23] Samovski D, Kalderon B, Yehuda-Shnaidman E, Bar-Tana J (2010). Gating of the mitochondrial permeability transition pore by long chain fatty acyl analogs *in vivo*. J Biol Chem.

[24] Junttila MR, Li SP, Westermarck J (2008). Phosphatase-mediated crosstalk between MAPK signaling pathways in the regulation of cell survival. FASEB J.

[25] Kim HS, Song MC, Kwak IH, Park TJ, Lim IK (2003). Constitutive induction of p-Erk1/2 accompanied by reduced activities of protein phosphatases 1 and 2A and MKP3 due to reactive oxygen species during cellular senescence. J Biol Chem.

[26] Foley TD, Petro LA, Stredny CM, Coppa TM (2007). Oxidative inhibition of protein phosphatase 2A activity: role of catalytic subunit disulfides. Neurochem Res.

[27] Li X, Huang Y, Jiang J, Frank SJ (2008). ERK-dependent threonine phosphorylation of EGF receptor modulates receptor downregulation and signaling. Cell Signal.

[28] Winograd-Katz SE, Levitzki A (2006). Cisplatin induces PKB/Akt activation and p38(MAPK) phosphorylation of the EGF receptor. Oncogene.

[29] Nakayama M, Sakai K, Yamashita A, Nakamura T, Suzuki Y, Matsumoto K (2013). Met/HGF receptor activation is regulated by juxtamembrane Ser985 phosphorylation in hepatocytes. Cytokine.

[30] Reuveni H, Flashner-Abramson E, Steiner L, Makedonski K, Song R, Shir A, Herlyn M, Bar-Eli M, Levitzki A (2013). Therapeutic destruction of insulin receptor substrates for cancer treatment. Cancer Res.

[31] Chung J, Uchida E, Grammer TC, Blenis J (1997). STAT3 serine phosphorylation by ERK-dependent and -independent pathways negatively modulates its tyrosine phosphorylation. Mol Cell Biol.

[32] Wakahara R, Kunimoto H, Tanino K, Kojima H, Inoue A, Shintaku H, Nakajima K (2012). Phospho-Ser727 of STAT3 regulates STAT3 activity by enhancing dephosphorylation of phospho-Tyr705 largely through TC45. Genes Cells.

[33] Mitsuhashi S, Shima H, Tanuma N, Sasa S, Onoe K, Ubukata M, Kikuchi K (2005). Protein phosphatase type 2A, PP2A, is involved in degradation of gp130. Mol Cell Biochem.

[34] Zheng Y, Xia Y, Hawke D, Halle M, Tremblay ML, Gao X, Zhou XZ, Aldape K, Cobb MH, Xie K, He J, Lu Z (2009). FAK phosphorylation by ERK primes ras-induced tyrosine dephosphorylation of FAK mediated by PIN1 and PTP-PEST. Mol Cell.

[35] Bryant P, Zheng Q, Pumiglia K (2006). Focal adhesion kinase controls cellular levels of p27/Kip1 and p21/Cip1 through Skp2-dependent and -independent mechanisms. Mol Cell Biol.

[36] Das Thakur M, Salangsang F, Landman AS, Sellers WR, Pryer NK, Levesque MP, Dummer R, McMahon M, Stuart DD (2013). Modelling vemurafenib resistance in melanoma reveals a strategy to forestall drug resistance. Nature.

[37] Sun C, Wang L, Huang S, Heynen GJ, Prahallad A, Robert C, Haanen J, Blank C, Wesseling J, Willems SM, Zecchin D, Hobor S, Bajpe PK (2014). Reversible and adaptive resistance to BRAF(V600E) inhibition in melanoma. Nature.

[38] Woods D, Parry D, Cherwinski H, Bosch E, Lees E, McMahon M (1997). Raf-induced proliferation or cell cycle arrest is determined by the level of Raf activity with arrest mediated by p21Cip1. Mol Cell Biol.

[39] Vredeveld LC, Possik PA, Smit MA, Meissl K, Michaloglou C, Horlings HM, Ajouaou A, Kortman PC, Dankort D, McMahon M, Mooi WJ, Peeper DS (2012). Abrogation of BRAFV600E-induced senescence by PI3K pathway activation contributes to melanomagenesis. Genes Dev.

[40] McDuff FK, Turner SD (2011). Jailbreak: oncogene-induced senescence and its evasion. Cell Signal.

